# Inhibition of DNA repair with MGMT pseudosubstrates: phase I study of lomeguatrib in combination with dacarbazine in patients with advanced melanoma and other solid tumours

**DOI:** 10.1038/bjc.2011.285

**Published:** 2011-08-02

**Authors:** H A Tawbi, L Villaruz, A Tarhini, S Moschos, M Sulecki, F Viverette, J Shipe-Spotloe, R Radkowski, J M Kirkwood

**Affiliations:** 1 Melanoma and Skin Cancer Program, University of Pittsburgh Cancer Institute, Pittsburgh, PA, USA; 2 Sarcoma Program, University of Pittsburgh Cancer Institute, Pittsburgh, PA, USA; 3 Division of Hematology/Oncology, Department of Medicine, School of Medicine, University of Pittsburgh, Pittsburgh, PA, USA; 4 Theradex Inc., Princeton, NJ, USA

**Keywords:** DNA repair, MGMT pseudosubstrate, lomeguatrib, melanoma, chemotherapy resistance

## Abstract

**Background:**

The DNA repair protein *O*^6^-methylguanine-DNA methyltransferase (MGMT) reverses the *O*^6^-methylguanine (*O*^6^-meG) lesion induced by dacarbazine. Depletion of MGMT can be achieved using *O*^6^-meG pseudosubstrates. Herein, we report the first phase I experience of the novel *O*^6^-meG pseudosubstrate lomeguatrib, combined with dacarbazine.

**Methods:**

This is a phase I dose-escalation study to determine the maximum tolerated dose and recommended phase II dose (RP2D) of lomeguatrib combined with a single dose of dacarbazine on a 21-day schedule.

**Results:**

The vast majority of the 41 patients enrolled had metastatic melanoma (36/41) and most had no previous chemotherapy (30/41). The most frequent non-hematological adverse events (AEs) were nausea (52%), and fatigue (42%). The most frequent AEs of grade 3–4 severity were neutropaenia (42%), leukopaenia (17%), and thrombocytopaenia (12%). Only 1 patient had a partial response and 10 patients had stable disease.

**Conclusion:**

The RP2D of lomeguatrib was 40 mg orally twice daily for 10 days combined with 400 mg m^−2^ of dacarbazine IV on day 2. Oral administration of lomeguatrib substantially increases the haematological toxicity of dacarbazine consistent with experience with other *O*^6^-meG pseudosubstrates.

The overall prognosis for patients with metastatic melanoma remains poor, with 5-year survival of 6% (Barth *et al*, 1995; Jemal *et al*, 2009; Rigel, 2010). Immunotherapy has recently achieved modest but significant improvements in survival in the metastatic setting; and mutation-directed targeted therapy is showing renewed promise (Flaherty *et al*, 2010; Hodi *et al*, 2010). However, despite those exciting advances, durable remissions are infrequent and the vast majority of metastatic melanoma patients progress and subsequently receive alkylator-based chemotherapy. Dacarbazine is the only approved chemotherapeutic agent for the treatment of metastatic melanoma, despite modest response rates (approximately 7–9%) in modern phase III trials (Middleton *et al*, 2000a; Tawbi and Kirkwood, 2007).

*O*^6^-methylguanine-DNA methyltransferase (MGMT) is a DNA repair protein and an established mechanism of resistance to alkylating agents. It is ubiquitously expressed, highly conserved, and vital to the maintenance of DNA integrity (Gerson, 2004). *O*^6^-methylguanine-DNA methyltransferase recognises the *O*^6^-methylguanine (*O*^6^-meG) base lesion induced by alkylating agents and transfers the methyl group to a cysteine residue in its active site. The guanine base is therefore repaired and can sustain regular replication and transcription, whereas the MGMT molecule is autoinactivated, ubiquitinated, and degraded (Liu and Gerson, 2006). Any additional DNA repair requires *de novo* synthesis of the protein (Pegg, 2000). This makes MGMT a target for inhibition as a resistance abrogation strategy (Spiro *et al*, 1999).

*O*^6^-methylguanine pseudosubstrates were developed with the goal of depleting MGMT by presenting it with decoy base lesions devoid of inherent toxicity, and therefore reversing chemotherapy resistance.

*O*^6^-(4-bromothenyl)-guanine (lomeguatrib, Patrin, KuDOS Pharmaceuticals, Ltd, Cambridge, UK) is an oral *O*^6^-meG pseudosubstrate. We performed a phase I study of lomeguatrib combined with dacarbazine in patients with advanced solid tumours.

## Materials and Methods

### Study design

This trial was a single-institution, dose-finding, phase I clinical trial whose primary objective was to determine the maximum tolerated dose (MTD) and recommended phase II dose (RP2D) of lomeguatrib when administered orally in combination with a single infusion of dacarbazine. The secondary objectives were to determine the dose-limiting toxicities (DLTs) and non-DLTs associated with the combination and to document the efficacy of the combination of lomeguatrib and dacarbazine in advanced solid tumours.

### Patients and methods

Patients were enrolled at the University of Pittsburgh Cancer Institute, between March 2004 and March 2007. Lomeguatrib was provided by KuDOS Pharmaceuticals, Ltd. All patients were ⩾18 years of age with histologically confirmed metastatic solid tumours refractory to standard therapy or for which no effective therapy was available. Patients were excluded from the study if they previously received therapy with dacarbazine.

### Drug administration

Patients were initially accrued on a 5-day schedule of administration (schedule A) and received lomeguatrib orally once daily on days 1 through 5 in combination with dacarbazine by short intravenous infusion on day 2 of a 21-day cycle (Table 1). The protocol was amended to allow for an extended dosing schedule for 10 days and at a fixed 40 mg twice daily dose that was shown to inhibit MGMT (schedule B). Patients on schedule B received lomeguatrib 40 mg PO BID for 10 days in combination with doses of dacarbazine on day 2 every 21 days, with provisions to de-escalate dacarbazine while keeping lomeguatrib at a fixed dose (Table 1).

### Toxicity and efficacy evaluations

Toxicity was assessed by clinical and laboratory examination and graded according to the Common Terminology Criteria for Adverse Events, version 3.0. Radiographic assessments of tumour status were done within 28 days of the start of cycle 1 and repeated every two cycles (every 6 weeks). Patients were evaluated for response using Response Evaluation Criteria in Solid Tumors (RECIST 1.0).

## Results

### Patient characteristics

Forty-one patients were treated in this study and were eligible for safety evaluations. The majority of patients (61%) had a baseline ECOG performance status of 1. The majority of patients (88%) had melanoma, either of cutaneous, uveal, or mucocutaneous origin. Two patients had colorectal carcinoma, and one patient each had oesophageal, small bowel, and small-cell lung cancer. A total of 11 patients had received previous chemotherapy, and 5 had no other previous therapies (Table 2).

### Dose-limiting toxicity/maximum tolerated dose

No DLTs were observed in the any of the first four cohorts on schedule A. One DLT was experienced in cohort 5 (dacarbazine 800 mg m^−2^ and lomeguatrib 80 mg once daily). In the first dosing cohort on schedule B (dacarbazine 700 mg m^−2^ and lomeguatrib 40 mg twice daily), there were two DLTs among four patients. The dose of dacarbazine was then reduced until the MTD was defined in cohort 9 (dacarbazine 400 mg m^−2^ and lomeguatrib 40 mg administered twice daily on days 1–10), in which one out of the first six patients enrolled experienced a DLT. This cohort was then expanded to nine patients, the ninth of whom experienced a DLT. Dose-limiting toxicities were most frequently due to grade 4 neutropaenia, grade 4 thrombocytopaenia, grade 3 and 4 leukopaenia, and grade 4 hyperuricaemia. Multiple dose modifications were required in subsequent cycles in almost all cohorts, although only one dose reduction was required in cohort 9. On the basis of these findings, the RP2D and schedule was dacarbazine on day 2 at 400 mg m^−2^ IV, with lomeguatrib 40 mg PO BID on days 1 through 10 of a 21-day cycle.

### Safety

All the 41 safety-evaluable patients experienced at least one drug-related adverse event (AE; Table 3). The most frequent grade 3–4 AEs were neutropaenia (42%), leukopaenia (17%), and thrombocytopaenia (29%). Other common AEs observed included nausea (51%), fatigue (42%), constipation (22%), and infusion site pain (22%).

In all, 19 (46%) of the 41 safety-evaluable patients had dose reductions due to AEs, 16 (40%) of whom had neutropaenia and 10 (24%) had thrombocytopaenia. Overall, 9 (22 %) of the 41 patients came off study due to AEs, which were most frequently neutropaenia (10%) and thrombocytopaenia (5%). The median number of cycles administered was two (range, one to nine cycles).

### Efficacy

A total of 38 patients were evaluable for efficacy: 1 patient (3%) had a partial response (PR), 10 patients (26%) had stable disease, and 27 patients (71%) had progressive disease after two cycles of therapy (Table 4). The one patient who achieved a PR had melanoma and a duration of response of 164 days. Stable disease was maintained as long as 204 days in a patient with melanoma, who went on to receive nine cycles of treatment on schedule A.

## Discussion

Overexpression of MGMT has an important role in tumour resistance to chemotherapy. *O*^6^-methylguanine-DNA methyltransferase is a DNA repair protein that does not activate a pathway, but instead recognises and repairs adducts at the *O*^6^ position of the guanine base in a suicidal stoichiometric reaction (Pegg *et al*, 1995). The inactivated protein is then ubiquitinated and degraded by proteasomes (Ayi *et al*, 1992; Smith *et al*, 1996).

Attempts to overcome the repair of *O*^6^-methylguanine adducts have focused on depletion and inhibition of MGMT. *O*^6^-methylguanine-DNA methyltransferase is depleted after exposure to alkylating agents, as the protein is consumed in repairing DNA damage. This was the basis for the development of low-dose extended schedules of TMZ administration, which has been shown to effectively deplete MGMT levels while permitting an almost two-fold greater level of exposure to the drug with minimal additional toxicity (Brock *et al*, 1998). A large phase III trial (EORTC 18032) in metastatic melanoma randomised 859 patients to receive TMZ 150 mg m^−2^ per day orally on days 1–7 repeated every 14 days (‘week on–week off’) or dacarbazine 1000 mg m^−2^ IV every 21 days, and showed no difference in overall survival and only a minor increase in response rates (10% *vs* 14% Patel *et al*, 2011). Compression of TMZ scheduling has also been evaluated with shorter dosing intervals such as twice a day in a phase I clinical trial that was not followed by a formal efficacy study (Middleton *et al*, 2000a; Spiro *et al*, 2001). In a phase II study, the 4-hourly schedule of TMZ was tolerated and led to higher response rates (23%) in melanoma (Middleton *et al*, 2000b).

Direct inhibition of MGMT using pseudosubstrates is attractive, as these agents are not in themselves toxic and can possibly lead to an improved therapeutic index.

*O*^6^-benzylguanine (*O*^6^-beG) was the first agent to reach clinical investigation and was used in combination with BCNU (carmustine), gliadel (BCNU in wafer form), and TMZ for treatment of different solid tumours, such as gliomas, melanomas, sarcomas, colon cancer, and lymphomas (Friedman *et al*, 1998; Spiro *et al*, 1999; Schilsky *et al*, 2000; Schold *et al*, 2004; Gajewski *et al*, 2005; Quinn *et al*, 2005; Warren *et al*, 2005; Weingart *et al*, 2007). Two phase I trials conducted at the University of Chicago (UC) and Case Western Reserve University (CWRU), evaluated toxicity in patients with advanced solid tumours or lymphoma. Patients received *O*^6^-beG intravenously, followed 1 h later by BCNU. The UC Trial determined that the MTD of BCNU when combined with 120 mg m^−2^ *O*^6^-beG was approximately 3-fold lower (40 mg m^−2^) than the standard clinical dose of BCNU (Schilsky *et al*, 2000). Increased haematological toxicity was the most significant AE associated with the addition of *O*^6^-beG to BCNU. In both studies, MGMT activity was successfully inhibited in peripheral blood mononuclear cells and even in tumour tissues in the CWRU Study (Spiro *et al*, 1999).

Increased myelosuppression continued to plague the development of this agent even in phase II trials; several patients with melanoma treated on a phase II trial of *O*^6^-beG and BCNU at 40 mg m^−2^ required additional dose reductions on the basis of haematological toxicity (Gajewski *et al*, 2005). This experience was reproduced in several phase II trials in other patient populations, such as soft tissue sarcoma, multiple myeloma, and glioblastoma multiforme (GBM), where the increased toxicity was not associated with comparable increases in efficacy (Quinn *et al*, 2002; Ryan *et al*, 2006; Batts *et al*, 2007). This outcome was attributed to the following factors: (a) MGMT levels rapidly recover within 24–48 h and (b) the total dose of alkylating agents delivered is curtailed by myelosuppression.

A phase I trial of TMZ (75 mg m^−2^) and lomeguatrib (40 mg) for 5 days was conducted by Middleton's Group in the United Kingdom and showed similar haematological toxicity and limited clinical efficacy, suggesting no advantage for this regimen over conventional TMZ administration in the treatment of melanoma (Ranson *et al*, 2006, 2007). A randomised phase II trial of this combination did not show increased efficacy despite increased toxicity over TMZ alone (Ranson *et al*, 2007). The dosing schedule of lomeguatrib was therefore extended to 10 days but did not improve efficacy (Kefford *et al*, 2009).

In this phase I study, lomeguatrib was administered with dacarbazine daily for 5 days and escalated to twice daily for 10 days. However, the MTD of dacarbazine was only 400 mg m^−2^, <50% of the standard (800–1000 mg m^−2^) clinical dose. Similar to the *O*^6^-beG experience, no clear signal of improved efficacy of dacarbazine was observed, although a formal phase II trial is yet to be conducted.

Promoter methylation of MGMT is a recognised predictor of improved response to TMZ-based chemotherapy in patients with GBM (Hegi *et al*, 2005). The role of MGMT as a predictive marker of response to alkylator-based chemotherapy in melanoma is much less defined, and MGMT may in fact be more valuable for the prediction of toxicity (Hassel *et al*, 2010).

The contribution of MGMT to melanoma resistance to methylating agents seems to be rather dependent on downstream pathways that are capable of recognising the persistent *O*^6^-guanine base damage and initiating apoptosis, such as the DNA mismatch repair pathway (MMR). Mismatch repair pathway deficiency leads to alkylator resistance regardless of MGMT levels in the cell, and thus makes MGMT inhibition less relevant. Mismatch repair pathway deficiency occurs frequently by epigenetic silencing through promoter methylation of key MMR proteins (hMLH1, PMS2, MSH2, and MSH6). In ovarian cell line models, it has been shown that reversal of MMR deficiency using hypomethylating agents restores the effect of MGMT inhibition on TMZ cytotoxiciy, validating this model. This concept was recently evaluated at our institution in a phase I/II clinical trial, in which TMZ was combined with the hypomethylating agent decitabine.

### Conclusion

The chemotherapy resistance of melanoma continues to be a significant challenge. Novel therapeutic agents targeting DNA repair have the potential to reverse this resistance. In this phase I study, the RP2D of lomeguatrib is 40 mg PO BID on days 1 through 10, with dacarbazine at 400 mg m^−2^ IV on day 2 of a 21-day cycle. Lomeguatrib significantly increases the toxicity of dacarbazine, similar to previous experience with MGMT pseudosubstrates. *O*^6^-methylguanine-DNA methyltransferase remains an interesting target for the modulation of chemotherapy resistance and may prove valuable in better patient selection for treatment with chemotherapy.

## Figures and Tables

**Table 1:** Dose-escalation scheme

**Schedule**	**Cohort**	**Dacarbazine (mg m^−2^)**	**Lomeguatrib (mg per day)**	**No. of patients**
A^a^	1	500	40	3
	2	500	80	3
	3	600	80	3
	4	700	80	3
	5	800	80	6
				
B^b^	6	700	80	4
	7	600	80	6
	8	500	80	3
	9	400	80	9

a Schedule A: once daily dosing of lomeguatrib on days 1–5 of each 21-day cycle.

b Schedule B: twice daily dosing of lomeguatrib on days 1–10 of each 2-day cycle.

**Table 2:** Characteristics of the 41 enrolled patients

**Characteristic**	**Schedule A**	**Schedule B**	**Total**
Number of patients	19	22	41
			
*Age, years*			
Median	54	54	54
Range	35–76	25–81	25–81
			
*Sex*			
Male	10 (53%)	13 (59%)	23 (56%)
Female	9 (47%)	9 (41%)	18 (44%)
			
*Ethnic origin*			
White	19 (100%)	21 (96%)	40 (98%)
Black	0	1 (4%)	1 (2%)
			
*ECOG performance* *status*			
0	6 (32%)	8 (36%)	14 (34%)
1	11 (58%)	14 (64%)	25 (61%)
2	1 (5%)	0	1 (2%)
Unknown	1 (5%)	0	1 (2%)
			
*Histopathology*			
Melanoma	14 (74%)	22 (100%)	36 (87%)
Adenocarcinoma, NOS	3 (16%)	0	3 (7%)
Mucinous adenocarcinoma	1 (5%)	0	1 (2%)
Small-cell carcinoma, NOS	1 (5%)	0	1 (2%)
			
*Previous therapy*			
Chemotherapy	7 (37%)	4 (18%)	11 (27%)
Immunotherapy	10 (53%)	13 (59%)	23 (56%)
Radiation	4 (21%)	4 (18%)	8 (20%)
Other	4 (21%)	1 (4%)	5 (12%)

Abbreviations: ECOG=Eastern Cooperative Oncology Group; NOS=not otherwise specified.

**Table 3:** Toxicities commonly observed (>20%) and grade 3–4 toxicities

	**All Patients (*N*=41)**		**Schedule B (*N*=22)**	**Schedule A (*N*=19)**
**Adverse event**	**All Grades**	**Grades 3–4**	**Grades 3–4**	**Grades 3–4**
Leukopaenia	8 (20%)	7 (17%)	4 (18%)	3 (16%)
Neutropaenia	19 (46%)	17 (41%)	11 (50%)	6 (32%)
Thrombocytopaenia	12 (29%)	5 (12%)	4 (18%)	1 (5%)
Nausea	21 (51%)	—	—	—
Fatigue	17 (41%)	—	—	—
Constipation	9 (22%)	—	—	—
Infusion site pain	9 (22%)	—	—	—

**Table 4:** Overall response^a^

	**Schedule A**	**Schedule B**	**Total**
No. of patients	19	19	38
Overall response (CR+PR)^b^	0	1 (5%)	1 (3%)
PR	0	1 (5%)	1 (3%)
SD	9 (47%)	1 (5%)	10 (26%)
PD	10 (53%)	17 (90%)	27 (71%)

Abbreviations: CR=complete response; PD=progressive disease; PR=partial response; RECIST=Response Evaluation Criteria in Solid Tumors; SD=stable disease.

a RECIST criteria.

b Overall response based on patients with either a CR or PR.
